# Updated World Health Organization Guideline on Preventing Early Pregnancy and Poor Reproductive Outcomes Among Adolescents in Low- and Middle-income Countries

**DOI:** 10.1016/j.jadohealth.2025.07.024

**Published:** 2025-11

**Authors:** Marina Plesons, Sheri Bastien, Ashok Dyalchand, Rajesh Mehta, Ilene S. Speizer, Venkatraman Chandra-Mouli

**Affiliations:** a Department of Public Health Sciences, University of Miami Miller School of Medicine, Miami, Florida; b Department of Sexual, Reproductive, Maternal, Child, Adolescent and Aging Health, World Health Organization, Geneva, Switzerland; c Institute of Health Management, Pachod, Aurangabad, India; d MAMTA Health Institute for Mother and Child, Public Health Foundation of India, New Delhi, India; e Department of Maternal and Child Health, University of North Carolina Gillings School of Global Public Health, Chapel Hill, North Carolina; f Independent Learner, Communicator, Advisor, Teacher, and Supporter of Adolescent Advocacy, Research, and Action, Geneva, Switzerland

Adolescent pregnancy remains a significant public health issue worldwide, with profound health, social, and economic consequences. In 2011, the World Health Organization (WHO) published its first guideline to address early pregnancy and poor reproductive outcomes among adolescents in developing countries [1], which aimed to provide policymakers and program managers with evidence-based interventions to reduce adolescent morbidity and mortality associated with early pregnancies. Recognizing the ongoing global challenge, WHO recently published an updated version of this guideline, reflecting over a decade of evolving evidence, shifting priorities, and emerging challenges [2].

## Context

Adolescent girls aged 15–19 years in low- and middle-income countries (LMICs) have an estimated 21 million pregnancies each year, 50% of which are unintended [3]. In 2021, an estimated 12.1 million girls aged 15–19 years and 499,000 girls aged 10–14 years gave birth globally [4]. Worldwide, the adolescent birth rate decreased from 64.5 births per 1,000 women aged 15–19 years in 2000 to 42.5 births per 1,000 women of the same age in 2021. However, rates of change have been uneven across different regions of the world [5,6]; likewise, there are substantial differences in adolescent pregnancy rates within regions and countries [5,7,8], with rates highest among girls with less education and/or of low economic status [9,10]. Adolescent pregnancies continue to be driven by a range of factors, including child marriage, limited educational and employment opportunities, child sexual abuse and intimate partner violence, and lack of access to comprehensive sexuality education and contraceptive services.

### What is similar and what is different in the updated guideline?

While many characteristics of the 2011 edition remain unchanged, the update of the WHO guideline on preventing early pregnancy and poor reproductive outcomes among adolescents in LMICs incorporates recent evidence and introduces several notable changes.

#### Rationale

The rationale for this guideline is similar to the 2011 edition of the guideline, namely that adolescent pregnancy is a significant public health issue worldwide with short- and long-term health, social, and economic consequences. However, given the progress made globally in reducing child marriage and increasing access to, uptake of, and continued use of contraception among adolescents, and given the shift in the field from addressing the needs of all adolescents to addressing the needs of groups of adolescents based on their particular needs and circumstances, there is a stronger focus on groups who have not benefited from this progress.

#### Objectives

The objective of the updated guideline is unchanged, namely to provide evidence-based normative guidance on interventions to improve adolescent morbidity and mortality by reducing the chances of early pregnancy and its resulting poor health outcomes.

#### Scope

The scope of the updated guideline is reduced. Given that there are separate guidelines which have been recently updated on four of the six outcomes included in the 2011 edition [[11], [12], [13], [14], [15], [16], [17]], this update focuses on the remaining two objectives: 1) preventing child marriage and responding to the needs and rights of married girls and 2) improving access to, uptake of, and continued use of contraception among adolescents.

#### Intended audience

The intended audience of the updated guideline is unchanged, namely, policy leaders/planners and program managers from government, nongovernmental organizations (NGOs), and agencies that provide technical and financial support in LMICs. Secondary audiences include health workers, researchers, government officials, professional associations, program managers, and advocacy groups. Finally, the guideline is also intended for adolescents, themselves.

#### Development process

The updated guideline was developed according to WHO standards and requirements for guideline development [18], with the oversight of the WHO Guidelines Review Committee. In identifying and prioritizing the Population, Intervention, Comparator, Outcome questions—which define the specific research questions used to guide the evidence reviews and formulation of recommendations—the Guideline Steering Committee applied the socioecological framework to ensure that individual, interpersonal, community and organizational, and social and policy-level factors influencing early pregnancy and poor reproductive outcomes among adolescents were adequately considered. All recommendations were developed by a Guideline Development Group, facilitated by a guideline methodologist using the Grading of Recommendations Assessment, Development, and Evaluation (GRADE) approach (see the Supplemental Material for more information; a full description of the methods is available in the guideline) [2]. One notable change was the involvement of teams representing different stakeholder groups—government, NGOs, academics, and young people—from one country in each of WHO's regions, as members of the External Review Group. In addition to the young people who were part of these six country teams, representatives of global networks/organizations working for and led by young people were included in the Guideline Development Group.

A second change is the inclusion of good practice statements, which may be issued when the quality of evidence for an intervention is low or very low, but when there is high certainty based on indirect evidence and/or expert opinion that the intervention does more benefit than harm and when not implementing the intervention would be contrary to practice norms. In terms of implementation, good practice statements should be viewed as equivalent to strong recommendations [18].

### What are the new recommendations and good practice statements?

**Figure 1:**
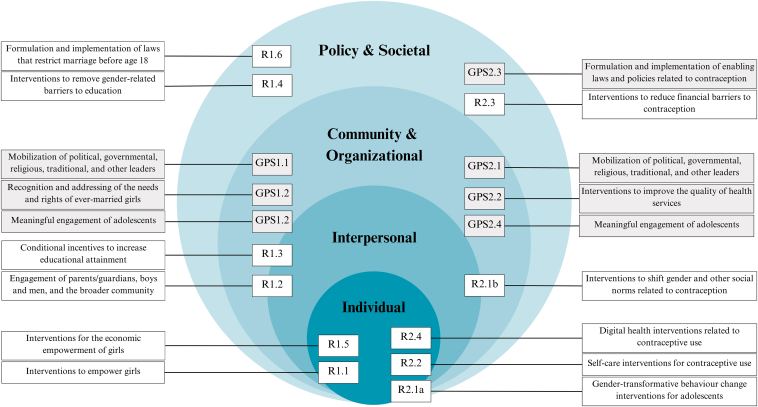
Recommendations and good practice statements by level of the social-ecological framework. R, recommendation; GPS, good practice statement.

The updated guideline includes 10 recommendations and seven good practice statements (see the Supplemental Material for a summary table), operating across different levels of the social-ecological framework (Figure 1).

#### Preventing child marriage and responding to the needs and rights of married girls

*Recommendation 1.1:* WHO recommends the implementation of interventions to empower girls by building their knowledge, skills, assets and social networks *(conditional recommendation; low-certainty evidence).*

WHO defines empowerment as a process through which people gain greater control over decisions and actions affecting their health [19]. While problem prevention approaches are important, there is growing recognition of the need for positive youth development approaches [20]. Research has demonstrated the importance of supporting adolescents' empowerment through opportunities to build core protective assets—competence, confidence, connection, character, and caring—which enable them to grow and develop in good health, avoid and mitigate poor health outcomes, and thrive in other aspects of their lives [[21], [22], [23]]. Interventions that hold the most promise are those that are girl-centered, engage the community as allies and advocates, build valued skills (e.g., financial literacy) as assets that cannot be taken away, create spaces where girls feel safe and supported, and connect girls to services [11,12,24].

*Recommendation 1.2:* WHO recommends that programs aiming to reduce child marriage and support married girls engage with parents/guardians, boys and men, and the broader community to create and sustain a gender-equitable and enabling environment *(conditional recommendation; low-certainty evidence).*

While building personal, social, and economic assets in adolescent girls is essential, it is often insufficient to create supportive and equitable environments. Such efforts must be combined with gender-synchronized approaches that engage men and women to challenge harmful norms and foster supportive environments [25,26]. Gender-synchronized approaches operate at the “intentional intersection of gender-transformative efforts reaching both men and boys and women and girls of all sexual orientations and gender identities. They engage people in challenging harmful and restrictive constructions of masculinity and femininity that drive gender-related vulnerabilities and inequalities and hinder health and well-being” [27]. In particular, men and boys have a role to play as partners in creating such a gender-equitable environment to benefit the lives of women and girls and their own. Given the complexity and nuance inherent to gender norms, the design and implementation of these interventions should be guided by an understanding of the structural underpinnings of the norms and gender stereotypes that they seek to shift [28].

*Recommendation 1.3:* WHO recommends offering conditional incentives (conditioned on school attendance and/or remaining unmarried) as a broad strategy to increase educational attainment and reduce child marriage as a part of social protection interventions for girls at highest risk of child marriage *(conditional recommendation; moderate-certainty evidence).*

Conditional incentives require specific actions, such as school attendance, while unconditional incentives are provided based on eligibility, with or without being labeled for a specific purpose [29,30]. Given that poverty is a major driver of child marriage—affecting 712 million people living below the US $2.15 per day poverty line in 2022—there has been growing interest in the use of such incentives to prevent child marriage [31]. Existing research evidence and programmatic experiences support their use to enrol girls in school, keep them in school, and ensure that they complete their schooling, as a means of preventing child marriage, among other outcomes [32].

*Recommendation 1.4:* WHO recommends the implementation of interventions to remove gender-related barriers to education and ensure girls' completion of 12 years of quality education *(strong recommendation; moderate-certainty evidence).*

While 50 million more girls were enrolled in school in 2023 compared with 2015, completion rates of lower and upper secondary education lag behind those of primary education [33]. Evidence shows that interventions addressing tuition costs, food provision, and academic support are most effective at addressing gender-related barriers to education, with promising results for those that improve water, sanitation, and school accessibility [34]. Achieving sustainable development goal 4, which aims for all girls and boys to complete 12 years of quality education by 2030, requires addressing these barriers while also ensuring the quality of educational opportunities [35].

*Recommendation 1.5:* WHO recommends the implementation of interventions aimed at the economic empowerment of girls to improve their financial literacy, access to savings, and employment skills and prospects, and to expand alternatives to marriage before age 18 years *(strong recommendation; moderate-certainty evidence).*

Evidence highlights that early marriage, early pregnancy, and low educational attainment are interconnected, and that delaying marriage and pregnancy while increasing education can enhance human capital, health outcomes, and empowerment [36]. Strategies such as vocational training, life skills development, mentoring, and access to job opportunities show promise in supporting economic empowerment and improving outcomes for adolescent girls, and in breaking the cycle of intergenerational poverty.

*Recommendation 1.6:* WHO recommends the formulation and implementation of laws that restrict marriage before age 18 years, consistent with human rights standards *(conditional recommendation; very-low-certainty evidence).*

Setting a legal minimum age of marriage is essential for protecting children from abuse, harm, violence, and exploitation, but laws alone are insufficient to eliminate child marriage. Likewise, criminalizing the practice can have unintended negative consequences for girls, their families, and their communities [37,38]. Comprehensive legal frameworks must address the root causes of child marriage, including gender inequality, and be accompanied by supportive policies and programs that respond to the drivers of the practice, contribute to social change, and strengthen systems. Effective implementation should involve consultations with civil society, center girls' rights and evolving capacities, and complement community-based efforts to challenge harmful norms and stereotypes.

*Good practice statement 1.1:* Political, governmental, religious, traditional, and other influential leaders should be mobilized to support the prevention of child marriage and the promotion of girls' rights.

Especially in contexts where marriage is viewed as a protective institution, building adolescent girls' personal, social, and economic assets is often insufficient to enable them to make autonomous decisions about marriage. As such, combining individual-level interventions with those at the community level is essential, as research shows influential leaders significantly shape family and community decisions [25,39]. What constitutes an influential leader varies by context and might include political, religious, and traditional leaders, business or thought leaders, youth influencers, athletes, actors, musicians, social media influencers, and others. Regardless, interventions should first understand leaders' interests, motivations, and perspectives as they pertain to child marriage and engage them with tailored messaging through multiple channels.

*Good practice statement 1.2:* Efforts to address the needs and rights of women and girls should recognize and address the specific needs and rights of ever-married girls and those in formal or informal unions.

Despite awareness of the harmful consequences of child marriage, married girls remain largely overlooked in policies and programs [40,41]. Existing interventions are often limited in their geographic reach and scope, typically focusing on sexual and reproductive health (SRH) while neglecting broader health, economic empowerment, education, legal rights, and agency [42,43]. In addition, maternal and newborn health programs rarely tailor services to adolescents, despite their unique needs [44]. There is thus an urgent need to integrate the needs and rights of ever-married girls and girls in formal or informal unions within interventions to address the needs and rights of girls and women, in general.

*Good practice statement 1.3:* Adolescents, including those who are ever married or in formal or informal unions, should be meaningfully engaged in the design, implementation, monitoring and evaluation of efforts to address their needs and rights.

Meaningful adolescent engagement is an inclusive, intentional, mutually-respectful partnership between adolescents and adults whereby power is shared, respective contributions are valued, and adolescents' ideas, perspectives, skills, and strengths are integrated into the design and delivery of policies and programs that affect their lives [45]. This approach goes beyond token participation, ensuring that adolescents are actively involved in decisions affecting themselves and their communities. Effective engagement is guided by principles of transparency, voluntariness, respect, and safety [45,46]. Embedding these principles into programs fosters more relevant, effective, and sustainable solutions that better address adolescents' diverse needs and rights.

#### Increasing access to, uptake of, and continued use of contraception among adolescents

*Recommendation 2.1a:* WHO recommends the implementation of gender-transformative behavior change interventions with adolescents to strengthen their ability to make decisions about their contraceptive use *(strong recommendation; moderate-certainty evidence).*

*Recommendation 2.1b:* WHO recommends the implementation of interventions to shift gender and other social norms to support contraceptive decision-making and access to, uptake of, and continued use of contraception among adolescents *(strong recommendation; moderate-certainty evidence).*

Social norms shape behaviors by defining what is perceived as acceptable, with deviations often met with social sanctions [47,48]. Gender norms, a subset of social norms, reinforce inequalities that privilege masculinity over femininity, undermining the rights of women and girls and limiting opportunities for women, men, and gender minorities to express their authentic selves [47,49]. Addressing these norms requires engaging parents, peers, partners, and the wider community to foster more supportive environments. Gender-transformative interventions, which address the root causes of gender inequality and shift power dynamics, are essential to achieving sustainable improvements in adolescent SRH and should be tailored to the specific gender and other social norms and contexts in which they are implemented [50,51].

*Recommendation 2.2:* WHO carried forward the recommendations in the WHO guideline on self-care interventions for health and well-being, 2022 revision that are relevant to adolescents' access to, uptake of, and continued use of contraception [52].

Self-care is defined as the ability to promote health, prevent disease, and cope with illness and disability independently, with or without a health-care provider [52]. Self-care interventions can increase autonomy, self-efficacy, and engagement in health decision-making while addressing common barriers that adolescents face when seeking contraceptive services, including stigma, discrimination, and lack of privacy [52,53]. Given the global health workforce shortage and persistent barriers faced by adolescents in accessing SRH care, expanding self-care interventions has the potential to improve contraceptive access and use among young people.

*Recommendation 2.3:* WHO recommends the implementation of interventions to reduce financial barriers related to access to, uptake of, and continued use of contraception among adolescents *(conditional recommendation; very-low-certainty evidence).*

Adolescents typically have fewer financial resources than adults; thus, the cost of services poses an important barrier, especially in settings where contraception is not subsidized or free [54]. While costs vary by context and contraceptive method, financial support interventions not only improve access but also increase demand for contraceptive services. Much of the available evidence focuses on voucher schemes; however, there is a need for context-specific approaches, as vouchers may not be feasible or appropriate in all settings.

*Recommendation 2.4:* WHO recommends the implementation of accurate and safe digital health interventions for adolescents as part of SRH programing *(conditional recommendation; low-certainty evidence).*

Digital health interventions have the potential to expand patient autonomy and reach of health services, and there is growing consensus that their strategic and innovative use will be an essential enabling factor to achieve universal health coverage and the 2030 Agenda for Sustainable Development. However, concerns remain about misinformation, data privacy, security, and exacerbated inequities due to the digital divide [[55], [56], [57], [58]]. As such, digital health interventions should be designed and implemented with careful consideration of equity, safety, and security and should complement, rather than replace, other approaches. Likewise, to prevent widening disparities, digital literacy should be integrated into broader health literacy initiatives [11].

*Good practice statement 2.1:* Political, governmental, religious, traditional and other influential leaders should be mobilized to support the access to, uptake of, and continued use of contraception among adolescents.

By leveraging leaders' influence in communities, interventions can help shift social norms, reduce stigma, and increase acceptance of adolescent contraceptive use. As previously noted, what constitutes an influential leader varies by context and might include political, religious, and traditional leaders, business or thought leaders, youth influencers, athletes, actors, musicians, social media influencers, and others. Regardless, interventions should first understand leaders' interests, motivations, and perspectives as they pertain to adolescent contraception and engage them with tailored messaging through multiple channels. Involving leaders with misconceptions or unsupportive attitudes could reinforce barriers; thus, in cases where sensitization is unsuccessful, it may be necessary to exclude certain leaders to avoid legitimizing restrictive views.

*Good practice statement 2.2:* Interventions to improve the quality of health services should be implemented to improve access to, uptake of, and continued use of contraception among adolescents.

WHO defines adolescent-friendly health services as those that are accessible, acceptable, equitable, appropriate, and effective [59]. However, evidence from high-, middle-, and low-income countries shows that adolescent services remain fragmented, poorly coordinated, and inconsistent in quality [60]. Ensuring high-quality services requires delivering contraceptive information and care in ways that respect adolescents' rights to dignity, autonomy, privacy, and confidentiality while addressing their unique needs [61]. This involves strengthening multiple key elements, including health literacy, community support, provider competencies, facility characteristics, and nondiscrimination [60]. Building on these principles, the concept of adolescent-responsive contraceptive services is a systems approach to institutionalize adolescent-responsive elements across the WHO health systems building blocks to improve access and quality of care for adolescents [54]. Doing so—across both public and private health sectors—is essential to ensure adolescents can make informed choices and access the care they need.

*Good practice statement 2.3:* Enabling laws and policies on age, marital status and consent procedures in relation to sexual activity, access to SRH services and access to specific contraceptive methods, should be coherently formulated and implemented to improve access to, uptake of, and continued use of contraception among adolescents.

States have a human rights obligation to ensure all individuals, including adolescents, have access to affordable, safe, and effective contraception [62]. However, age and marital status restrictions continue to limit access in many countries [63]. In addition, legal exceptions and contradictory laws and policies create confusion, deterring adolescents from seeking care and discouraging health providers from offering services [64]. Removing restrictive policies and ensuring legal clarity are critical to enabling adolescents to exercise their reproductive rights and access the contraception they need.

*Good practice statement 2.4:* Adolescents should be meaningfully engaged in the design, implementation, monitoring, and evaluation of efforts to address their contraceptive needs and rights.

As previously described, meaningful adolescent engagement is an inclusive, intentional, mutually-respectful partnership between adolescents and adults whereby power is shared, respective contributions are valued, and adolescents' ideas, perspectives, skills, and strengths are integrated into the design and delivery of policies and programs that affect their lives [45]. This approach goes beyond token participation, ensuring that adolescents are actively involved in decisions affecting themselves and their communities. Effective engagement is guided by principles of transparency, voluntariness, respect, and safety [45,46]. Embedding these principles into programs fosters more relevant, effective, and sustainable solutions that better address adolescents' diverse needs and rights.

### How should policy-makers and program managers select evidence-based interventions to respond to the needs of their specific context?

Selecting evidence-based interventions to prevent early pregnancy and poor reproductive outcomes among adolescents requires a context-specific, data-driven approach. The Global Accelerated Action for the Health of Adolescents guidance outlines a step-by-step process to tailor interventions effectively, with the involvement of relevant stakeholders, including adolescents [65].

The first step is to set priorities by conducting: [1] a needs assessment to identify the most pressing adolescent health concerns, including disparities by age, sex, and vulnerability; [2] a landscape analysis of existing policies, programs, and resources to assess gaps and opportunities, and [3] a consultative and inclusive priority-setting exercise based on public health impact, feasibility, and potential to address underserved populations.

The second step is to choose from a menu of proven interventions to address the drivers of early pregnancy and poor reproductive outcomes in a particular context. Alongside frameworks such at the Population Council's categorization of child marriage drivers and the International Center for Research on Women's framework of demand and supply side barriers to adolescent contraceptive use, the updated guideline provides a set of effective interventions based on recent evidence to consider for contextualized implementation at national and subnational levels [25,66].

The third step is to consider how to optimally balance global goals with local needs. Adopting the HIV “know your epidemic, tailor your response” approach can help policy-makers identify intervention “hotspots” and avoid common pitfalls like overcomplication and insufficient evidence use [67]. The updated guideline provides evidence-informed interventions that can be selected, combined, and adapted based on the specific needs, contexts, and capacities of different settings. The interventions need to address the behaviours of adolescents and the gatekeepers and the underlying determinants of those behaviours to achieve the desired health outcomes. Behaviours, determinants, and interventions logic models setting out how each recommendation/good practice statement will contribute to the desired outcomes have been provided (see Supplementary Material for logic models related to improving access to, uptake of and continued use of contraception, and reducing the levels of child marriage and improving responses to the health and social needs of ever-married girls). In addition, equity-focused approaches, such as WHO's Inequality monitoring in sexual, reproductive, maternal, newborn, child and adolescent health and the Innov8 framework, can be used to ensure that marginalized groups are not left behind in intervention planning [68,69].

Finally, monitoring and evaluation should follow structured frameworks, such as the International Health Partnership Common Monitoring and Evaluation Framework, to assess progress across inputs, outputs, and impact indicators, ensuring interventions remain effective and sustainable over time [70,71].

### Next steps

#### Plans for dissemination

Awareness and interest in the updated guideline will be promoted via traditional and social media, targeted emails, briefings, peer-reviewed articles, and conference presentations. Capacity building efforts will include in-person and virtual seminars highlighting the recommendations, their evidence base, and practical applications across country contexts. Direct support to countries will be provided through partnerships—such as Family Planning 2030 and Girls Not Brides—to demonstrate context-specific implementation. These efforts will be coordinated with WHO's regional and country offices; United Nations partners including the United Nations Population Fund (UNFPA)–United Nations Children’s Fund Global Program to End Child Marriage and the Global Partnership Forum on comprehensive sexuality education; and other academic, professional, and NGO collaborators.

#### Plans for addressing evidence gaps

While many studies have been published since the first edition of the guideline, significant evidence gaps remain due to unaddressed issues, inadequate study designs, or incomplete reporting. These limitations reduced the number and strength of studies eligible for the GRADE process. Identified gaps will inform ongoing research priority-setting exercises. Future studies should improve disaggregation by adolescent age groups and enhance attribution of effects through robust designs—such as factorial designs, mediation analyses, and detailed reporting of intervention components—to enable inclusion in future GRADE assessments.

#### Plans for future guideline updates

As for the 2011 guideline, decisions on future updates will be based on assessment of need. Future updates will follow WHO's standards and requirements for guideline development, including the possibility of utilizing a living guideline approach [72].

## Conclusion

The updated WHO guideline on preventing early pregnancy and poor reproductive outcomes among adolescents in LMICs is a timely and important resource that reflects the latest evidence and evolving global priorities. It serves as both a practical tool for immediate action to strengthen national policies and strategies for impact at the country level, and a call to reinforce global commitments to adolescent SRH and rights to drive long-term improvements in gender equality, education, and economic opportunities and to contribute to broader Sustainable Development Goals. It also underscores critical gaps in the evidence base and highlights the need for continued research to strengthen future guidance and implementation.
