# Supplementary material for: Updated World Health Organization Guideline on Preventing Early Pregnancy and Poor Reproductive Outcomes Among Adolescents in Low- and Middle-income Countries
Source: J Adolesc Health. 2025 Nov;77(5):803–9. doi: 10.1016/j.jadohealth.2025.07.024 (PMC12586972; doi:10.1016/j.jadohealth.2025.07.024)
Supplement: Supplementary Material [file mmc1.docx]

**Supplemental Material**

Summary of recommendations and good practice statements

| **1. Preventing child marriage and responding to the needs and rights of married girls** | |
| --- | --- |
| Recommendation 1.1 | WHO recommends the implementation of interventions to empower girls by building their knowledge, skills, assets and social networks.  *(Conditional recommendation; low-certainty evidence)* |
| Recommendation 1.2 | WHO recommends that programmes aiming to reduce child marriage and support married girls engage with parents/guardians, boys and men, and the broader community to create and sustain a gender-equitable and enabling environment.  *(Conditional recommendation; low-certainty evidence)* |
| Recommendation 1.3 | WHO recommends offering conditional incentives (conditioned on school attendance and/or remaining unmarried) as a broad strategy to increase educational attainment and reduce child marriage as a part of social protection interventions for girls at highest risk of child marriage.  *(Conditional recommendation; moderate-certainty evidence)* |
| Recommendation 1.4 | WHO recommends the implementation of interventions to remove gender-related barriers to education and ensure girls’ completion of 12 years of quality education.  *(Strong recommendation; moderate-certainty evidence)* |
| Recommendation 1.5 | WHO recommends the implementation of interventions aimed at the economic empowerment of girls to improve their financial literacy, access to savings, and employment skills and prospects, and to expand alternatives to marriage before age 18.  *(Strong recommendation; moderate-certainty evidence)* |
| Recommendation 1.6 | WHO recommends the formulation and implementation of laws that restrict marriage before age 18, consistent with human rights standards.  *(Conditional recommendation; very-low-certainty evidence)* |
| Good practice statement 1.1 | Political, governmental, religious, traditional and other influential leaders should be mobilized to support the prevention of child marriage and promotion of girls’ rights. |
| Good practice statement 1.2 | Efforts to address the needs and rights of women and girls should recognize and address the specific needs and rights of ever-married girls and those in formal or informal unions. |
| Good practice statement 1.3 | Adolescents, including those who are ever married or in formal or informal unions, should be meaningfully engaged in the design, implementation, monitoring and evaluation of efforts to address their needs and rights. |
| **2. Increasing access to, uptake of, and continued use of contraception among adolescents** | |
| Recommendation 2.1a | WHO recommends the implementation of gender-transformative behaviour change interventions with adolescents to strengthen their ability to make decisions about their contraceptive use.  *(Strong recommendation; moderate-certainty evidence)* |
| Recommendation 2.1b | WHO recommends the implementation of interventions to shift gender and other social norms to support contraceptive decision-making and access to, uptake of, and continued use of contraception among adolescents.  *(Strong recommendation; moderate-certainty evidence)* |
| Recommendation 2.2 | WHO carried forward the recommendations in the *WHO guideline on self-care interventions for health and well-being, 2022 revision* that are relevant to adolescents’ access to, uptake of, and continued use of contraception (52). These recommendations include:  Self-administered injectable contraception should be made available as an additional approach to deliver injectable contraception for individuals of reproductive age.  *(Strong recommendation; moderate-certainty evidence)*  Over-the-counter oral contraceptive pills (OCPs) should be made available without a prescription for individuals using OCPs.  *(Strong recommendation; very-low-certainty evidence)*  Over-the-counter emergency contraceptive pills should be made available without a prescription to individuals who wish to use emergency contraception.  *(Strong recommendation; moderate-certainty evidence)*  The consistent and correct use of male and female condoms is highly effective in preventing the sexual transmission of HIV; reducing the risk of HIV transmission both from men to women and women to men in serodiscordant couples; reducing the risk of acquiring other sexually transmitted infections and associated conditions, including genital warts and cervical cancer; and preventing unintended pregnancy.  Provide up to one year’s supply of pills, depending on the woman’s preference and anticipated use. Programmes must balance the desirability of giving women maximum access to pills with concerns regarding contraceptive supply and logistics. The resupply system should be flexible, so that the woman can obtain pills easily in the amount and at the time she requires them. |
| Recommendation 2.3 | WHO recommends the implementation of interventions to reduce financial barriers related to access to, uptake of, and continued use of contraception among adolescents.  *(Conditional recommendation; very-low-certainty evidence)* |
| Recommendation 2.4 | WHO recommends the implementation of accurate and safe digital health interventions for adolescents as part of sexual and reproductive health (SRH) programming.  *(Conditional recommendation; low-certainty evidence)* |
| Good practice statement 2.1 | Political, governmental, religious, traditional and other influential leaders should be mobilized to support the access to, uptake of, and continued use of contraception among adolescents. |
| Good practice statement 2.2 | Interventions to improve the quality of health services should be implemented to improve access to, uptake of, and continued use of contraception among adolescents. |
| Good practice statement 2.3 | Enabling laws and policies on age, marital status and consent procedures in relation to sexual activity, access to SRH services and access to specific contraceptive methods, should be coherently formulated and implemented to improve access to, uptake of, and continued use of contraception among adolescents. |
| Good practice statement 2.4 | Adolescents should be meaningfully engaged in the design, implementation, monitoring and evaluation of efforts to address their contraceptive needs and rights. |

Description of the guideline development process

The updated guideline was developed according to WHO standards and requirements for guideline development, based on the *WHO handbook for guideline development, second edition*, and with the oversight of the WHO Guidelines Review Committee (18).

All of the recommendations in the guideline were developed by the Guideline Development Group (GDG), facilitated by the guideline methodologist using the Grading of Recommendations Assessment, Development and Evaluation (GRADE) approach (73). For a description of how the specific recommendations and good practice statements included in this guideline were determined, please refer to Annex 3 of the guideline.

The guideline was updated using a step-by-step process, as set out by WHO. First, consultations were held with the intended audience of the 2011 edition of the guideline to determine whether they believed an update would be useful to inform the work they were doing. Second, a stock-taking review was conducted to determine whether there were publications in the public arena to respond to the research questions on adolescent contraceptive use. This was not needed on child marriage because two major reviews had recently been carried out (74, 75). Third, partnerships were forged with United Nations Population Fund (UNFPA) and United States Agency for International Development (USAID), with which WHO developed the 2011 edition of the guideline. Fourth, the groups of key contributors to the guideline development process were constituted: a Guideline Steering Group, a Guideline Development Group (GDG), an External Review Group (ERG) comprising small teams from one country in each of WHO’s six regions (Argentina in the Region of the Americas, Bangladesh in the South-East Asia Region, Burkina Faso in the African Region, the Republic of Moldova in the European Region, the Philippines in the Western Pacific Region and Yemen in the Eastern Mediterranean Region), and two systematic review teams (one for research questions on child marriage and the other for research questions on adolescent contraceptive use) along with an experienced methodologist. Fifth, in consultation with the Guideline Steering Group and the GDG as well as other stakeholders, the PICO questions were developed in an open and consultative manner. Sixth, a series of GDG meetings were held – in July 2022 (virtual), March 2023 (virtual), June 2023 (in person), July 2023 (virtual) and August 2023 (virtual) – at which the process was set out; respective roles were defined; PICO questions were reviewed and finalized; evidence and the Evidence-to-Decision tables were presented and discussed; and recommendations were formulated. Alongside the meetings of the GDG, country-level consultations were supported in the six countries mentioned above to draw upon the inputs of a wider range of stakeholders. Seventh, the updated edition of the guideline was drafted and reviewed by the GDG, the Guideline Steering Group, and the ERG. Eighth, the updated edition of the guideline was reviewed and approved by the Guidelines Review Committee. Finally, the guideline was published and plans to disseminate it and support its application were operationalized.

**Behaviours, determinants, and interventions logic model setting out how each recommendation/good practice statement will contribute to the desired outcome of improving access to, uptake of and continued use of contraception by adolescents**

| **Interventions recommended** | **Attributes proposed in the Guideline for the recommendation to be successful.** | **Strengthened protective factors and weakened risk factors as a result of the interventions (across all levels of a socio-ecological framework).** | **Adolescent behaviours directly related to the health outcome** | **Intermediate outcome** |
| --- | --- | --- | --- | --- |
| Recommendations | | | | |
| R1a. Implementing gender-transformative behaviour change interventions with adolescents to strengthen their ability to make decisions about their contraceptive use. | Interventions are tailored to the specific gender and other social norms and contexts in which they are implemented.  Interventions address the ways in which such norms differentially impact sub-groups of adolescents. | Community level: Leaders and influential members in general, and men and boys in particular acknowledge and accept the rights of girls/women to make decisions about contraceptive use (including both shared and individual ones) and follow up on these decisions.  Individual level: Adolescents have the knowledge, skills and confidence to discuss contraceptive use, to negotiate its use, and to contribute to/make decisions on its use. | Adolescents discuss contraceptive use with service providers, their partners, and other influential people in their immediate context.  They negotiate with partners to use contraceptives.  They contribute to shared decision making, or make their own decisions, when appropriate. | Increase in the access to, uptake and continued use of contraception by adolescents. |
| R.1b. Implementing interventions to shift gender and other social norms to support contraceptive-decision making and access to, uptake of, and continued use of contraception among adolescents. | Efforts are targeted at leaders and other influential members to understand and to challenge and change restrictive attitudes and norms on gender in general and on sexuality and contraceptive use by adolescents in particular.  Efforts are made to shift these attitudes and norms to make them more gender equitable and accepting of adolescent sexuality.  These efforts are tailored to the specific gender and other social norms and contexts in which they are implemented. | Societal level/Community level: Leaders and other influential persons acknowledge that adolescent girls face barriers in making decisions about using contraceptives and in using them (and that some of these barriers are based on patriarchal attitudes and norms about girls’/women’s rights to make decisions about their lives including their sexual lives).  They contribute to creating an environment that is supportive of adolescents obtaining and using contraception.  Family members and partners sense the shift towards attitudes and norms supportive of contraceptive access, uptake of and use by adolescents, and support it themselves.  Individual level: Adolescents sense the supportive attitudes and norms. | Community leaders/members, partners and other influential family members, and contraceptive service providers support decision making, access to, uptake of and continued use of contraception by adolescents. | Increase in the access to, uptake of and continued use of contraception by adolescents. |
| R2. Self-administered injectable contraception should be made available as an additional approach to deliver injectable contraception for individuals of reproductive age.  Over-the-counter oral contraceptive pills should be made available without a prescription for individuals using oral contraceptive pills.  Over-the-counter emergency contraceptive pills should be made available without a prescription to individuals who wish to use emergency contraception. | Self-care opportunities are implemented / strengthened and complemented with efforts to ensure the availability of the full range of modern methods of contraception. | Societal level: Laws and policies support the provision of contraceptives mentioned in the recommendations for adolescents, without the prescription / approval / involvement of a health worker.  Community level: The contraceptives mentioned in the recommendations are provided within and outside health facilities, without requiring a health worker’s prescription/approval/involvement.  Individual level: Adolescents are aware that they can legally obtain the contraceptives mentioned in the recommendation from health facilities and other delivery points in their communities. | Adolescents obtain contraceptives as and when they want to from different sources in their communities and use them as a result of expanded access accessibility. | Increase in the access to, uptake of and continued use of contraception by adolescents. |
| R3. Interventions to reduce financial barriers related to access to, uptake of, and continued use of contraception among adolescents. | Measures to remove or reduce costs for adolescents, as appropriate. | Societal level: Laws and policies to provide contraceptives to adolescents at no cost/reduced cost from different service delivery points are formulated and applied.  Community level: Leaders and members are aware of the laws and policies, and assist health facility staff in applying them, with support where possible.  Individual level: Adolescents are aware that they can obtain contraceptives at reduced price or at no charge, and where and how to get them. | Adolescents obtain the contraceptives they need from different sources and use them. | Increase in the access to, uptake of, and continued use of contraception by adolescents. |
| R4. Accurate and safe digital health interventions for adolescents as part of sexual and reproductive health programming. | Digital health interventions are delivered in combination with other health interventions to promote contraceptive use by adolescents.  Digital health interventions for adolescents are designed taking into account cognitive capacity, equity, accuracy of content, and safety (notably data privacy). | Community level/societal level: Access to digital health mechanisms is made easier to adolescents.  Individual level: Girls and boys are confident in using digital health options to get information about contraception, and where/how to get them.  As a result, they are motivated to use contraception, and feel confident to obtain and use contraception. | Adolescents use digital health options confidently and safely to obtain accurate information about contraceptives they need and about different sources to obtain and use them. | Increase in the access to, uptake and continued use of contraception by adolescents. |
| Good practice statements | | | | |
| GP1 Political, governmental, religious, traditional, and other influential leaders are mobilized to support the access to, uptake of, and continued use of contraception by adolescents | Influential leaders in each setting are carefully identified. They may include political governmental, religious and traditional leaders as mentioned in the good practice statement. In addition – depending on the context, they may include business or thought leaders, youth influencers, athletes, actors, musicians, social media influencers and others.  Their perspectives, interests, and motivation are sought and taken into account in framing messages and targeting them.  If leaders have unsupportive attitudes and practices, and are unwilling/unlikely to change, them, a decision may need to be made not to engage with them. | Societal level/Community level: Efforts targeted at influential leaders contribute to an environment that is supportive of adolescents obtaining and using contraception. | Adolescents obtain the contraceptives they need from different contraception delivery points and use them. | Increase in the access to, uptake of and continued use of contraception by adolescents |
| GP2 Interventions to improve the quality of health services should be implemented to improve access to, uptake of, and continued use of contraception by adolescents. | Health services for adolescents meet local iterations of global standards on health literacy, community support, appropriate packages of services, provider competencies, facility characteristics, equity and non-discrimination, data and quality improvement, and participation.  Contraceptive information and services are provided in a way that ensures fully informed decision making, respects dignity, autonomy, privacy and confidentiality, and is sensitive to individual’s needs and perspectives. | Societal level: Laws and policies require the provision of high-quality adolescent friendly services including contraceptive information and services to adolescents from a variety of delivery points. Adequate resources are allocated and technical guidance provided for this to be done.  Community level: Community leaders and members support the provision of contraceptive information and services to adolescents, and for their uptake by adolescents.  Family level: Family members too support this.  Health system level: Managers and service providers take steps to understand and overcome barriers to service use. Adolescents are meaningfully involved in these efforts.  Individual level: Adolescents are aware of where, when and how to obtain the contraceptive information and services they need. | Adolescents obtain the contraceptives they need easily from different contraceptive delivery points including health facilities and use them. | Increase in the access to, uptake and continued use of contraception by adolescents |
| GP 3 Enabling laws and policies on age, marital status, and consent procedures in relation to sexual activity, access to sexual and reproductive health services and access to specific contraceptive methods, should be coherently formulated and implemented to improve access to, uptake of and continued use of contraception among adolescents. | Laws and policies are harmonized and do not contain discrepancies.  They are formulated and implemented in line with human rights standards, with the objective of assuring health and wellbeing rather than punishment.  The laws and policies are not implemented in isolation. Instead, they are complemented with other interventions recommended in the guideline. | Society level: There is widespread awareness of the laws and their rationale. There is also support for it.  Community level: Community leaders and members are aware of the laws and their rationale. They are supportive of it.  Individuals: Adolescent girls and boys are aware of the laws and policies and that they have shaped their own attitudes and norms in the community. | Adolescents obtain the contraceptives they need from different sources and use them. | Increase in the access to, uptake of and continued use of contraception by adolescents |
| GP 4 Adolescents should be meaningfully engaged in the design, implementation, monitoring and evaluation of efforts to address their contraceptive needs. | The meaningful engagement of adolescents is ensured across the programme cycle, but particular attention is paid to ensuring it in the design stage and in monitoring and evaluation for accountability purposes using a human-rights based approach. | Societal level: There is awareness and growing acceptance and support, for the government’s requirement of meaningful engagement of adolescents in initiatives to meet their contraceptive needs.  Community level: Community leaders and members also share this awareness and sentiments, and support efforts in the community.  Family level: Families also share this awareness and sentiments, and support the adolescents in their families to engage with/contribute to local initiatives.  Individual level: Adolescents are aware of and feel this support. They engage in and contribute to local initiatives. | Adolescents contribute to the design, implementation, monitoring and evaluation of initiatives to meet their contraceptive needs, thereby making them more responsive to adolescents’ needs. | Increase in the access to, uptake of and continued use of contraception by adolescents |

Example of indicators and means of verification

| **Interventions recommended** | **Attributes proposed in the Guideline for the recommendation to be successful.** | **Strengthened protective factors and weakened risk factors as a result of the interventions** | **Adolescent behaviours directly related to the health outcome** | **Intermediate outcome** |
| --- | --- | --- | --- | --- |

| GP2 Interventions to improve the quality of health services should be implemented to improve access to, uptake of, and continued use of contraception by adolescents. | Health services for adolescents meet local iterations of global standards on health literacy, community support, appropriate packages of services, provider competencies, facility characteristics, equity and non-discrimination, data and quality improvement, and participation.  Contraceptive information and services are provided in a way that ensures fully informed decision making, respects dignity, autonomy, privacy and confidentiality, and is sensitive to individual’s needs and perspectives. | Societal level: Laws and policies require the provision of high-quality contraceptive information and services to adolescents from a variety of delivery points. Adequate resources are allocated for this to be done.  Community level: Community leaders and members support the provision of contraceptive information and services to adolescents, and for their uptake by adolescents.  Family level: Family members also support this.  Individual level: Adolescents are aware of where, when and how to obtain the contraceptive information and services they need. | Adolescents obtain the contraceptives they need from different contraceptive delivery points including health facilities. . | Increase in the access to, uptake of and continued use of contraception by adolescents |
| --- | --- | --- | --- | --- |
|  | Indicator: Proportion of service delivery points that meet the stipulated standards of quality contraceptive information and service provision. Means of verification: Health facility quality assessment. | Indicator: Whether national laws and policies adhere to 10 key elements of WHO’s recommendations on quality adolescent health services and fulfils human rights.  Means of verification: Assessment of national laws and policies. | Indicator: Proportion of subgroup of adolescents (e.g., those of a particular age and social status) who obtain contraceptives from specific service delivery points.  Means of verification: (i) Exit interview (ii) Coverage survey. (iii) Service Register (in case of health facilities). | Indicator: Proportion of subgroup of adolescents (e.g., those of a particular age and social status) who report contraceptive use, and sustained contraceptive use.  Means of verification: Coverage Survey. |

**Behaviours, determinants, and interventions logic model setting out how each recommendation/good practice statement will contribute to the desired outcome of reducing the levels of child marriage and improving responses to the health and social needs of ever-married girls.**

| **Interventions recommended** | **Attributes proposed in the Guideline for the recommendations and good practice statements to be successful.** | **Strengthened protective factors and weakened risk factors as a result of the interventions (across different levels of an ecological framework)** | **Adolescent behaviours directly related to the health outcome** | **Intermediate outcome** |
| --- | --- | --- | --- | --- |
| Recommendations | | | | |
| R 1 Building girls’ knowledge, skills, assets and social networks. | Initiatives that are girl-centered, engage with the community as allies and advocates, build valued skills as assets that cannot taken away (e.g., financial literacy), create spaces where girls feel safe and supported, and connect girls to services.  Initiatives that include comprehensive sexuality education. | Individual level: Girls have the knowledge, skills and confidence to resist pressure to get married as children. They are aware of individuals and institutions around them whom they could turn to for support, and feel able to do so when needed. | Girls advocate for themselves and seek the support of others to advocate for them to avoid child marriage. | Reduction in the levels of child marriage |
| R. 2 Engaging with parents/guardians, boys and men, and the broader community to create and sustain a gender-equitable and enabling environment. | Initiatives seek to understand and address the structural underpinnings of norms and gender stereotypes.  Note: In some settings, this might include transactional aspects of marriage (e.g., the practice of bride price/dowry), especially in the context of socioeconomic hardship, and how these aspects drive decisions about marriage timing. In others settings, this might include norms and gender stereotypes about girls going to school and about women’s entry into the workforce. In settings where premarital sex and pregnancy are drivers of child marriage, changes in norms are brought about by greater acknowledgement of adolescent sexual activity (coerced and/or consensual) and better access to sexual and reproductive health services to prevent pregnancy. | Parents/guardians, boys and men, and other community members support girls to consider and embark on life trajectories other than child marriage. | Girls feel supported by the influential people around them to embark on life choices other than child marriage. | Reduction in the levels of child marriage. |
| R 3 Providing conditional incentives (conditioned on school attendance and/or remaining unmarried) for girls at the highest risk of child marriage. | Incentives are conditioned rather than unconditioned.  Incentive schemes use school attendance as a proxy for remaining unmarried because that is easier to verify and possible to enforce.  Incentive schemes are of a long duration and provide the possibility of future incentives.  Incentive schemes are designed and executed with the express objective of reducing inequity, and not increasing it.  Incentive schemes are not discontinued without adequate preparation. | Societal level: There is support for efforts to assist needy families to keep their girls in school and avoid child marriage.  Community level: This support extends to the community as well.  Family level: Families have an economic incentive to keep their girls in school and unmarried. | Girls stay enrolled in school. | Reduction in levels of child marriage. |
| R.4 Interventions to remove gender-related barriers to education and ensure girls’ completion of 12 years of quality education. | Interventions address all adolescents but prioritize the most vulnerable ones.  Girls who drop out of school, including due to marriage or pregnancy, are permitted/encouraged/supported to re-enter education, including alternatives to formal education.  The overall effort also addresses boys and aims to ensure that they also get the support they need to complete at least 12 years of education. | Societal/Community levels: There is awareness and growing acceptance of/support for efforts to overcome barriers to girls’ continuing schooling, even after pregnancy and childbirth.  Families level: Families face fewer obstacles in supporting girls to continue with their schooling.  Individual levels: Obstacles to girls – especially the most marginalized ones - continuing/completing schooling are removed. | Girls stay enrolled in school or return to school if they have dropped out. | Reduction in levels of child marriage, and in the case of girls who dropped out of school because they were married/pregnant to prevent rapid repeat pregnancies. |
| R 5 Interventions to empower girls economically by improving their financial literacy, access to savings, and employment skills and prospects. | Initiatives are in place to build livelihood skills, including financial literacy.  Alongside this, initiatives are in place to provide information and support on employment opportunities, include self-employment. | Societal/community levels: Girls are seen as economic agents, and there is support for investing in their capacity to become economically active.  Family level: Girls are not seen as a financial burden. Instead, they are seen as assets.  Individual level: Girls feel supported and are able to find employment, including self-employment, and enabled to see pathways other than child marriage in their lives. | Girls are engaged in economically gainful activities. | Reductions in levels of child marriage. |
| R 6 The formulation and implementation of laws that restrict marriage before 18; consistent with human rights standards. | Laws are formulated after wide consultation.  Laws on child marriage are harmonized with other relevant laws.  The implementation of laws is combined with an active public information/education programme, that includes girls and boys.  It is also accompanied with programmes to support marginalized communities who might resort to child marriage in the context of insecurity or economic distress.  When sanctions follow the breaking of the law, they are not harsh and are combined with social measures. | Society/Community level: There is widespread awareness of the laws and their rationale, and for the accompanying social programmes. There is also support for it.  Community level: Community leaders and members are aware of the laws and their rationale and accompanying social programmes. Because of the likely consequences of the legal measures and social sanctions, they are not supportive of child marriage.  Family level: Families are aware of the laws and the implications of breaking them.  Individuals: Girls and boys are aware of the laws and about how they have shaped social norms. They also are aware that they could seek help if they are pressured to get married as children. | Girls resist efforts to have them married before the stipulated legal age. | Reduction in levels of child marriage. |
| Good practice statements | | | | |
| GP 1 Political, governmental, religious, traditional, and other influential leaders are mobilized to support the prevention of child marriage and the promotion of girls’ rights. | Influential leaders in each setting are carefully identified. In every setting, this includes female leaders as well.  Their perspectives, interests, and motivation are taken into account in framing messages and in targeting leaders.  If leaders have unsupportive attitudes and practices, and are unwilling/unlikely to change them, they are not engaged to support the programme. | Societal level: Attitudes and norms support the prevention of child marriage, and opposition for it.  Community level: Attitudes and norms support the prevention of child marriage, and opposition for it.  Family level: Families that want to avoid marrying their girls as children feel supported by the prevailing attitudes and norms. Those who are inclined to practice it, are hesitant to do so.  Individual level: Girls and boys know and feel the prevailing sentiment against child marriage. | Girls are not married before the stipulated legal age | Reduction in levels of child marriage |
| GP 2 Efforts to address the needs and rights of women and girls recognize and address the specific needs and rights of ever-married girls and those in formal or informal unions. | Existing initiatives to respond to the health and social needs of women and girls are tailored to take into account the particular health and social needs and problems of ever-married girls.  The health initiatives include but go beyond sexual and reproductive health. | Societal level: There is awareness that ever-married girls face health and social problems, and that initiatives are being put in place to respond to them.  Community level: Community leaders and members are also aware of this and are supportive of initiatives to respond to ever-married girls in their community.  Family level: Families are also aware of this, and that ever-married girls in their families can get the help and support they need from service providers in the community. They facilitate care seeking.  Individual level: Ever-married girls are also aware of this. They enrol in initiatives and seek care when they need to, with the support of influential family members around them. | Ever-married girls and their families are proactively targeted in existing health and social programmes to meet the needs and fulfil the rights of women and girls. | Reduction in health and social problems adversely affecting ever-married girls and their children. |
| GP 3 Adolescents, including those who are married or in formal or informal unions, are meaningfully engaged in the design, implementation, monitoring and evaluation of efforts to address their needs and rights. | Initiatives to prevent child marriage and respond to ever-married girls engage them in line with the following principles: (i) rights-based (ii) transparent and informative (iii) voluntary and free from coercion (iv) respectful of their views, backgrounds and identities, and (v) safe. | Societal level: There is awareness and growing acceptance and support, for the government’s requirement for the meaningful engagement of adolescents in child marriage initiatives.  Community level: Community leaders and members also share this awareness and sentiments, and support efforts in this area, in their communities.  Family level: Families also share this awareness and sentiments and support the adolescents in their families to engage with/contribute to local initiatives.  Individual level: Adolescents are aware of and feel empowered to engage in and contribute to local initiatives. | Adolescent girls and boys contribute to the design, implementation, monitoring and evaluation of initiatives to prevent child marriage and respond to the needs of ever-married girls. | Reduction in levels of child marriage, and fulfilment of the needs of ever-married girls. |

Example of indicators and means of verification

| **Interventions recommended** | **Attributes proposed in the Guideline for the recommendation to be successful.** | **Strengthened protective factors and weakened risk factors as a result of the interventions** | **Adolescent behaviours directly related to the health outcome** | **Intermediate outcome** |
| --- | --- | --- | --- | --- |
| 1.5 Interventions to empower girls economically by improving their financial literacy, access to savings, and employment skills and prospects. | Initiatives are in place to build livelihood skills, including financial literacy.  Alongside this, initiatives are in place to provide information and support on employment opportunities, include self-employment. | Societal level: Girls are seen as economic agents, and investing in their capacity to become economically active are supported.  Community level: Girls are seen as economic agents, and investing in their capacity to become economically active are supported.  Family level: Girls are not seen as a financial burden. Instead, they are seen as assets.  Individual level: Girls are able to find employment, including self-employment, and so can see pathways other than early marriage in their lives. | Girls are engaged in economically gainful activities,  they do not get married early. | Reductions in levels of child marriage. |
| 1.5 Interventions to empower girls economically by improving their financial literacy, access to savings, and employment skills and prospects. | Indicator: Proportion of subgroup of girls (e.g., those of a particular age and of a particular social-economic group) who have completed a particular financial literacy building intervention.  Means of verification: (i) Records maintained by the three largest providers of such interventions in the area. (ii) Survey of the girls. | Indicator: Proportion of male heads of a subgroup of families (e.g., those of a particular age and of a particular social-economic group) who believe that their daughters are more likely to be gainfully employed than girls like them five years ago. Means of verification: Survey | Indicator: Proportion of subgroup of girls (e.g., those of a particular age and of a particular social-economic group) who are in gainful employment.  Means of verification: Survey. | Indicator: Proportion of subgroup of girls (e.g., those of a particular age and of a particular social-economic group) who are in gainful employment.  Means of verification: Survey. |
